# Supplementary material for: Seminoma and Embryonal Carcinoma Footprints Identified by Analysis of Integrated Genome-Wide Epigenetic and Expression Profiles of Germ Cell Cancer Cell Lines
Source: PLoS One. 2014 Jun 2;9(6):e98330. doi: 10.1371/journal.pone.0098330 (PMC4041891; doi:10.1371/journal.pone.0098330)
Supplement: File S1 — ZIP file containing DMRforPairs output for significant regions. Please start from the html files. (ZIP) [file pone.0098330.s008.zip › figures/20049.pdf]

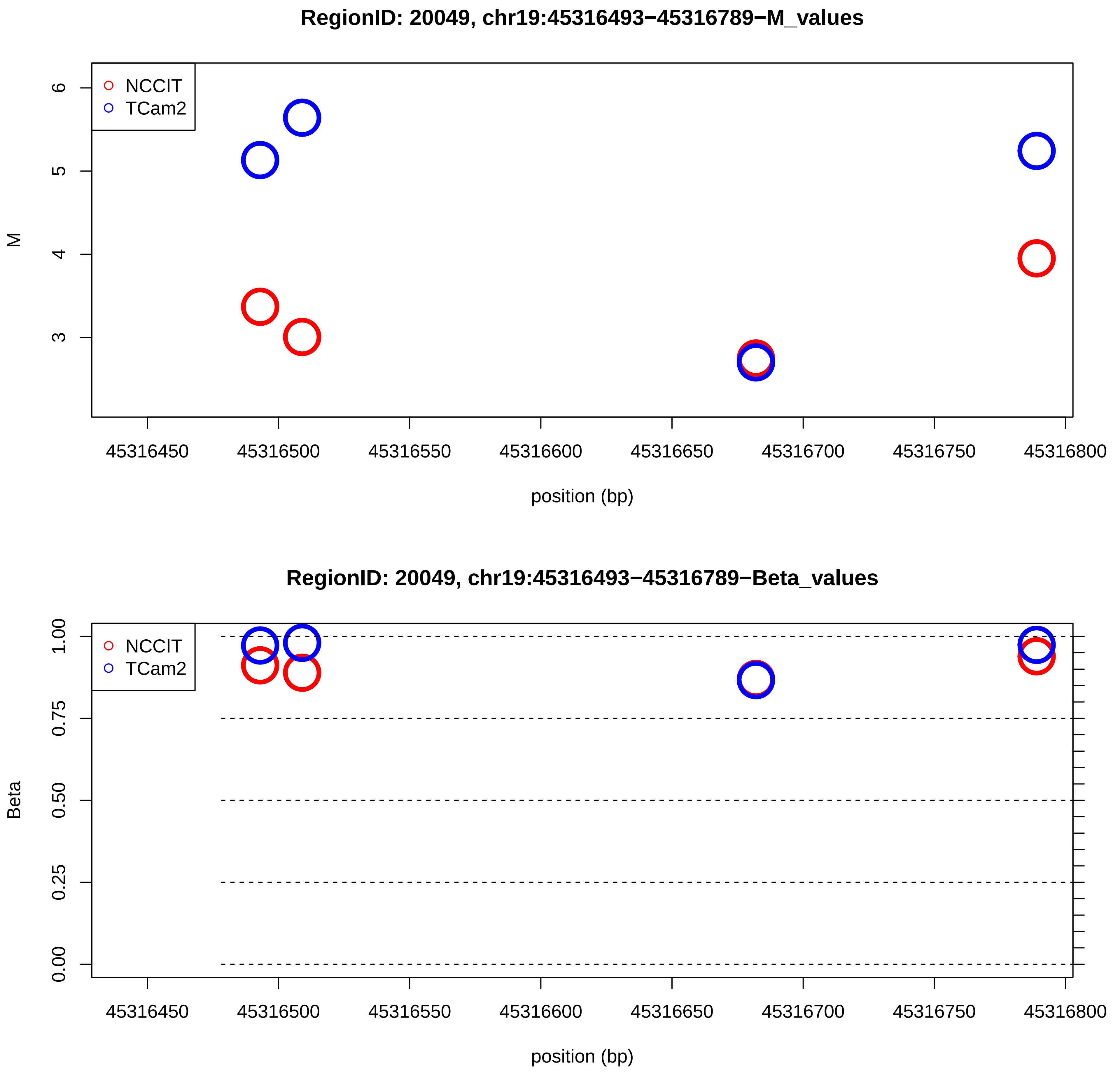
RegionID: 20049, chr19:45316493−45316789−M_values
M
position (bp)
NCCIT
TCam2
3
4
5
6
45316450
45316500
45316550
45316600
45316650
45316700
45316750
45316800
RegionID: 20049, chr19:45316493−45316789−Beta_values
Beta
position (bp)
NCCIT
TCam2
0.00
0.25
0.50
0.75
1.00
45316450
45316500
45316550
45316600
45316650
45316700
45316750
45316800
